# Trichoscopic Markers as Predictors of Treatment Response in Androgenetic Alopecia: A Systematic Review

**DOI:** 10.1111/jocd.71193

**Published:** 2026-09-15

**Authors:** Monalisa Manik, Nelva Karmila Jusuf, Dedy Hermansyah, Imam Budi Putra

**Affiliations:** ^1^ Department of Dermatology and Venereology, Faculty of Medicine Universitas Sumatera Utara Medan Indonesia; ^2^ Department of Surgical Oncology, Faculty of Medicine Universitas Sumatera Utara Medan Indonesia

**Keywords:** androgenetic alopecia, dermoscopy, hair regrowth, treatment response, trichoscopy

## Abstract

**Background:**

Androgenetic alopecia (AGA) is the most common form of hair loss in adults characterized by follicular miniaturization and hair thinning. Common treatments include topical minoxidil and oral finasteride. Accurate, noninvasive diagnostic tools such as trichoscopy are essential for guiding therapy and predicting treatment response.

**Aims:**

To evaluate key trichoscopic markers—including Hair Shaft Diameter Variability (HSDV), Terminal‐to‐Vellus Hair Ratio (T/V ratio), and vellus hair percentage—and their correlation with disease severity and therapeutic outcomes in AGA.

**Methods:**

A systematic review was conducted on studies published from 2021 to 2026 that quantitatively assessed trichoscopic markers for predicting treatment response in AGA. Due to heterogeneity in study methodologies, a qualitative synthesis was performed.

**Results:**

Elevated HSDV reflects the coexistence of terminal and miniaturized hairs, with reductions after treatment indicating reversal of miniaturization and serving as an early sensitive marker of efficacy. The T/V ratio increases with effective treatment, mirroring follicular health improvement. Changes in vellus hair percentage provide indications of active disease progression or recovery.

**Conclusion:**

Trichoscopic markers offer valuable frameworks for personalized treatment and clinical monitoring. Integration of such markers into routine practice is supported to optimize the management of AGA.

## 1 Introduction

Androgenetic alopecia (AGA) is the most common form of hair loss in adults and is characterized by progressive follicular miniaturization and increased hair shaft diameter variability, which lead to visible thinning and reduced hair density. AGA has a substantial epidemiological burden and important psychosocial impact, motivating widespread use of topical and systemic therapies such as topical minoxidil and oral finasteride [1, 2, 3]. Accurate, noninvasive diagnostic and monitoring tools are therefore essential to guide treatment choice and to predict therapeutic response.

Trichoscopy (dermoscopy of the scalp) has emerged as a practical in‐office tool to characterize hair changes in AGA. Systematic reviews and cohort studies show that the most consistent trichoscopic features in AGA include hair shaft diameter variability (HSDV), an increased proportion of vellus hairs, the peripilar sign, and miniaturization patterns; these features correlate with disease stage and can be monitored longitudinally [4, 5]. Importantly, several studies suggest that quantitative trichoscopic parameters—particularly HSDV and the terminal‐to‐vellus (T/V) hair ratio—can predict response to standard therapies (topical minoxidil, oral finasteride) and may help identify patients more likely to benefit from a given treatment [4, 5, 6, 7, 8].

Despite growing evidence, there remains heterogeneity in measurement methods, thresholds, and reporting across studies. Recent large‐scale epidemiologic data highlight the variable prevalence and presentation of AGA in different populations and underscore the need for reproducible, validated trichoscopic predictors that can be applied in routine clinical practice [1]. This manuscript therefore aims to (1) summarize the current evidence on trichoscopic predictors of therapeutic response in AGA, (2) propose a pragmatic set of baseline trichoscopic parameters (including HSDV and T/V ratio) for clinical use, and (3) discuss implications for personalized treatment selection and future research.

## 2 Materials and Methods

**FIGURE 1:**
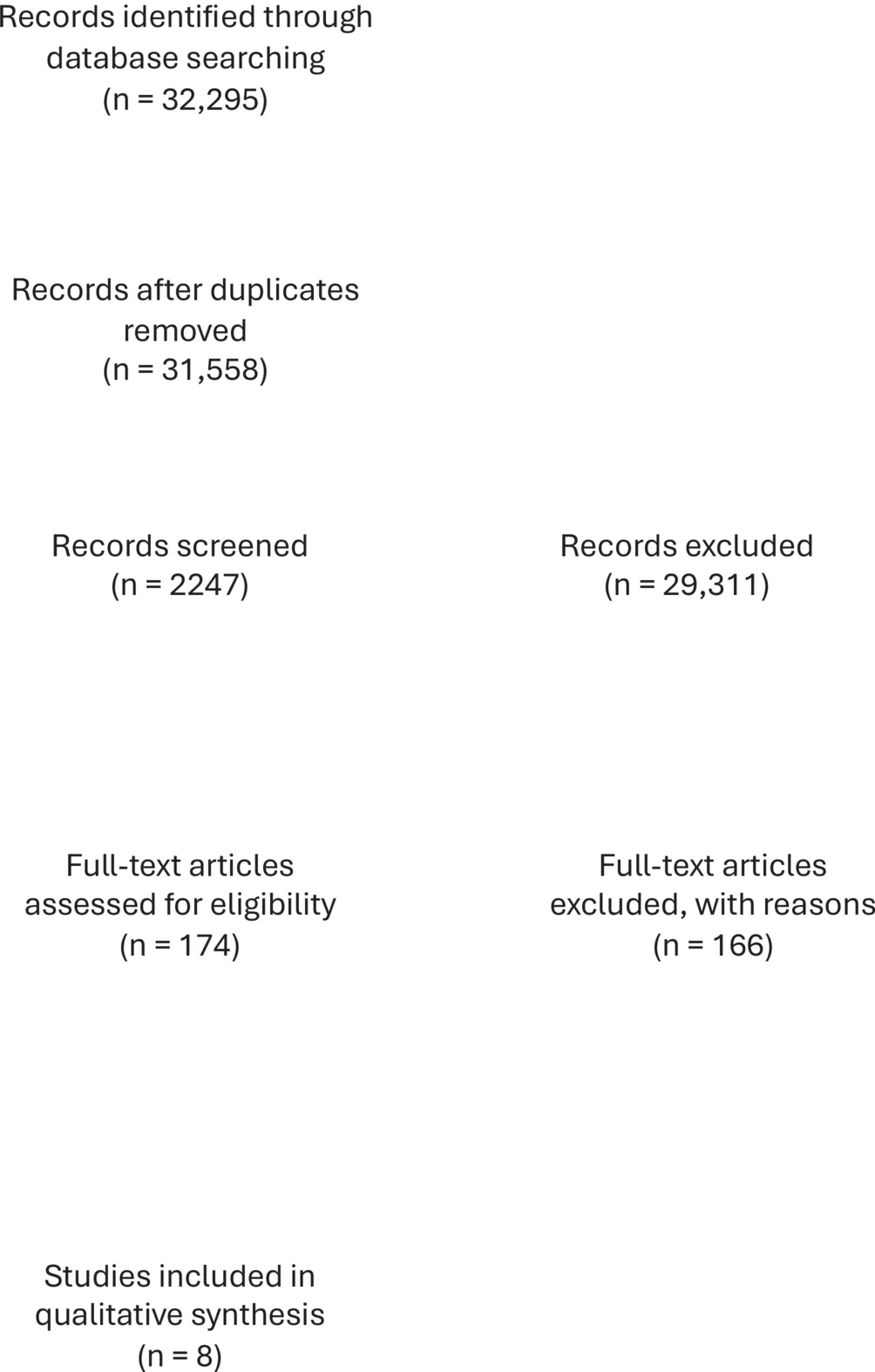
Preferred Reporting Items for Systematic Reviews and Meta‐Analyses (PRISMA) statement of the systematic review.

This research employs a systematic review approach following the Preferred Reporting Items for Systematic Reviews and Meta‐analysis (PRISMA) guidelines for systematic reviews (Figure 1). This method is systematically executed, adhering to correct research steps and protocols. A systematic review is a method that classifies and categorizes previously generated evidence through a structured series of evaluation, review, and classification. The systematic review procedure involves thorough preparation and sequencing, distinguishing it significantly from methods that merely provide literature studies.

We searched MEDLINE (via PubMed), Embase, Scopus, and Web of Science from January 1, 2021 to March 31, 2026 for original studies, prospective cohorts, randomized trials, and imaging‐driven analyses reporting trichoscopic or dermoscopic markers correlated with treatment outcomes in AGA. The primary search string combined terms for disease, imaging modality, and outcomes: (“androgenetic alopecia” OR “female pattern hair loss” OR “male pattern hair loss” OR AGA) AND (trichoscopy OR dermoscopy OR dermatoscopy OR “hair videodermoscopy” OR “digital phototrichogram”) AND (treatment OR therapy OR minoxidil OR finasteride OR “hair regrowth” OR “treatment response” OR “outcome”).

### 2.1 Study Selection

Two independent reviewers screened the titles and abstracts of records identified by database searches and manual reference checks; differences were resolved by consensus. Full texts were retrieved for records that passed the initial screen or when eligibility was unclear. The pool of candidate records included all primary studies that fulfilled the eligibility criteria.

Inclusion criteria to be considered were peer‐reviewed original research (randomized controlled trials, prospective cohorts, longitudinal observational studies, and case–control studies) that reported quantitative or semi‐quantitative trichoscopic/dermoscopic markers (e.g., hair shaft diameter variability [HSDV], terminal‐to‐vellus ratio [T/V ratio], hair density, vellus percentage, perifollicular signs) and related these markers to treatment outcomes in adult patients with AGA (18–65 years old); Studies examining topical minoxidil, oral 5‐α‐reductase inhibitors, or other pharmacologic interventions where trichoscopic measures were used longitudinally to predict/regard response; Publications in English between 2021 and 2026 (date limits reflect the focus of the manuscript).

Exclusion criteria involved case reports/series with < 10 patients, conference abstracts without full text, reviews (except used for reference mining), non‐human studies, and studies with mixed alopecia types where AGA‐specific data could not be disaggregated.

### 2.2 Data Synthesis

Given methodological heterogeneity (differences in trichoscopic device, measurement thresholds, outcome definitions, and timepoints), we first synthesized results qualitatively, focusing on consistency of directional effects for primary markers (HSDV, T/V ratio), and time‐to‐detection of early changes.

### 2.3 AI Disclosure

ChatGPT was used for summarizing original research data, language arrangement, grammar checking, and clarifying phrasing. The AI outputs were reviewed, edited, and verified by the author.

## 3 Results

### 3.1 Hair Shaft Diameter Variability (HSDV) as a Primary Marker of Miniaturization Reversal

Hair shaft diameter variability (HSDV) has emerged as a pivotal quantitative marker in assessing the degree of follicular miniaturization and its potential reversal during treatment of androgenetic alopecia (AGA). Elevated HSDV reflects the coexistence of thick terminal hairs and thinner miniaturized hairs within the same scalp area, indicating dynamic follicular activity. Several studies demonstrate that a reduction in HSDV after therapeutic intervention, such as topical minoxidil or oral finasteride, correlates with a favorable response characterized by the thickening and normalization of hair shafts, suggesting reversal of miniaturization [9, 10]. For example, Kim et al. showed that patients exhibiting significant reduction in HSDV after 24 weeks of topical minoxidil treatment had an increased proportion of terminal hairs, indicating rejuvenated follicular function [11].

Furthermore, trichoscopic analysis quantifying HSDV provides a sensitive and noninvasive method to monitor early treatment efficacy beyond mere hair counts, which can lag clinically. HSDV variability also serves as a predictive biomarker in precision medicine approaches for alopecia, allowing personalized therapeutic adjustments based on the degree of variability and miniaturization evident in baseline assessments [12]. These findings underscore the clinical utility of HSDV not only as a diagnostic measure but also as a dynamic marker reflecting ongoing follicular remodeling during treatment.

### 3.2 Terminal‐To‐Vellus Hair Ratio (T/V Ratio): Dynamic Indicator of Follicular Conversion

The terminal‐to‐vellus hair ratio (T/V ratio) is a fundamental trichoscopic metric reflecting the balance between mature terminal hairs and thin, miniaturized vellus hairs. This ratio dynamically changes in response to follicular conversion, where terminal follicles deteriorate into vellus‐like types in AGA progression. Multiple clinical investigations illustrate that a low T/V ratio is characteristic of active miniaturization and disease severity in patients with AGA [13, 14]. For instance, a cohort study evaluating patients before and after finasteride therapy demonstrated a statistically significant increase in the T/V ratio concomitant with clinical and photographic improvements, confirming its role as a surrogate endpoint for follicular improvement [15].

In addition, the T/V ratio serves as a longitudinal marker to monitor therapeutic efficacy. Transition from a diminished T/V ratio toward normalization indicates reversion of follicular miniaturization and restoration of hair density. As the ratio increases, it reflects enhanced follicular health and terminal hair regrowth, which remains the ultimate therapeutic goal in AGA management [16]. This ratio, thus, provides insight into the dynamic balance of follicular fate and is invaluable for clinicians employing trichoscopy in routine practice.

### 3.3 Vellus Hair Percentage and Its Role in Identifying Active Miniaturization

Vellus hair percentage quantification is critical in distinguishing active miniaturization zones from stable or resolved areas in hair loss disorders. An increased vellus hair percentage signifies ongoing follicular miniaturization and correlates with histopathological evidence of follicular regression [17, 18]. In active phases of androgenetic alopecia, this percentage rises due to the transformation of terminal follicles into short, thin vellus hairs. For example, trichoscopic evaluations performed in a longitudinal study indicated that patients with higher baseline vellus hair percentages were more likely to exhibit disease progression unless treated aggressively [19].

This parameter complements the T/V ratio and HSDV by offering a direct measure of the miniaturized hair population. It is especially useful in differentiating androgenetic alopecia from other non‐scarring alopecias where vellus hair changes may not be as prominent [20]. Therapeutic response is often marked by a decrease in vellus hair percentage, reflecting suppression of miniaturization and potential follicular regeneration, which is documented in treatment trials with topical and systemic agents [4]. Thus, vellus hair percentage is a valuable quantitative marker for diagnosing active disease and assessing response.

### 3.4 Perifollicular Signs and Inflammatory Markers

Perifollicular signs, notably perifollicular erythema and scaling, serve as clinical indicators of perifollicular inflammation frequently accompanying follicular miniaturization in AGA. Histological and trichoscopic studies reveal an inflammatory infiltrate around affected follicles marked by lymphocytic cells, which may contribute to follicular damage and disease progression [16, 21]. For instance, trichoscopic perifollicular scaling and redness were significantly more prevalent in patients with progressive AGA compared to controls, suggesting an active inflammatory process [22].

Emerging evidence positions these inflammatory markers as both diagnostic and prognostic indicators. Treatments targeting inflammation, in addition to anti‐androgenic therapies, have shown greater efficacy in halting disease progression and promoting follicular recovery. Monitoring perifollicular signs via trichoscopy enables clinicians to tailor combination therapies for multifactorial disease control. Moreover, elevated inflammatory cytokines detected in scalp biopsies correlate with clinical severity, reinforcing the importance of incorporating inflammatory assessment into therapeutic algorithms [19].

### 3.5 Treatment Response in Androgenetic Alopecia (AGA)

Table 1 synthesizes findings from recent clinical and observational studies on the use of dermoscopy (trichoscopy) to predict and monitor treatment response in androgenetic alopecia (AGA). The data spans a wide range of therapies, including minoxidil, finasteride, combination therapies, and emerging AI‐driven approaches. The table highlights how noninvasive dermoscopic assessments can detect early biological changes that precede visible clinical improvement, making them powerful tools for early decision‐making and personalized treatment.

**TABLE 1:** Findings from recent clinical and observational studies.

Author (Year)	Study design	Therapy evaluated	Dermoscopic parameters assessed	Main findings related to treatment response
Kuczara et al. (2024)	Systematic Review	Multiple (minoxidil, finasteride)	Hair shaft diameter diversity, perifollicular pigmentation, yellow dots, vellus hair ratio	Highlighted the value of hair diameter diversity and vellus hair increase as indicators of miniaturization and response to therapy. Trichoscopy is a reliable non‐invasive method for monitoring treatment response in AGA.
İritaş and Özcan (2024)	Prospective Observational	Various topical therapies including minoxidil and finasteride	Hair density, thickness, perifollicular erythema, anisotrichosis	Trichoscopy effectively tracked treatment response; increases in hair density and reduction in anisotrichosis (variation in hair shaft diameter) correlated with clinical improvement.
Rossi and Caro (2024)	Prospective Randomized Controlled Trial	Combination topical minoxidil + finasteride versus monotherapy	Hair density, hair shaft diameter diversity, perifollicular pigmentation	Combination therapy showed superior improvement in hair density and normalization of dermoscopic markers versus monotherapy, supporting synergistic effects observable via trichoscopy.
Johnson et al. (2025)	Retrospective Service Evaluation	Combined oral minoxidil and finasteride	Trichoscopic parameters detailed but not standardized	Clinical and trichoscopic improvements noted, including increased fiber diameter and hair density. Suggests oral combination therapy may enhance microstructural hair changes detected via dermoscopy.
Sinclair et al. (2025)	Observational Study	Sublingual minoxidil	Hair fiber diameter as proxy for follicle miniaturization	Sublingual minoxidil increased fiber diameter, suggesting reversal of miniaturization detectable by trichoscopy, making fiber diameter a valuable and sensitive parameter in treatment monitoring.
Shukla et al. (2026)	Prospective Cohort Study	Topical minoxidil	Early changes in hair shaft diameter and density	Early trichoscopic changes, such as increased hair shaft thickness and reduced vellus hair ratio, predicted favorable treatment response, supporting trichoscopy as an early monitoring tool.
Zengarini et al. (2026)	Review with Computational Models	Various including AI‐driven analysis	Automated quantification of hair density, diameter, and scalp health indicators	Emerging AI and computational tools improve precision and objectivity in trichoscopic evaluation; these methods show promise for personalized treatment monitoring in AGA.
Feldman et al. (2023)	Systematic Review with Meta‐analysis	Multiple therapies	Various trichoscopic markers indirectly assessed across studies	Found treatment efficacy correlated with reversal of miniaturization signs like diameter variability and increased hair count; resistance linked to persistent dermoscopic abnormalities.

The therapeutic landscape of androgenetic alopecia is guided by objective metrics including changes in HSDV, T/V ratio, vellus hair percentage, and inflammatory signs. Clinical trials consistently show that FDA‐approved treatments such as topical minoxidil and oral finasteride induce improvements in these parameters, reflecting follicular regeneration and disease control [10, 15, 23]. Individualized treatment response evaluation via trichoscopy allows early detection of responders versus non‐responders, optimizing therapy duration, and combinations. Moreover, novel therapeutic agents targeting inflammatory pathways present promising adjuncts to traditional therapies. The integration of quantitative trichoscopic markers as endpoints in clinical practice and trials facilitates precision medicine in trichiatry, improving prognostication and patient outcomes. Overall, treatment response in AGA is best assessed through a multimodal approach combining morphological and inflammatory markers for comprehensive management [12, 16].

## 4 Discussion

### 4.1 Rethinking the Role of Trichoscopy in AGA Management

Trichoscopy has gained prominence as a non‐invasive, reliable diagnostic modality in androgenetic alopecia (AGA) management. Traditionally considered an adjunctive tool, trichoscopy now offers detailed visualization of hair and scalp features that directly correlate with the pathophysiology of AGA. Kuczara et al. [4] demonstrated that trichoscopic parameters such as hair shaft diameter diversity and perifollicular pigmentation allow precise differentiation between AGA and other hair loss disorders, improving diagnostic accuracy. Moreover, the ability to repeatedly monitor these parameters has made trichoscopy a cornerstone in longitudinal management.

The dynamic nature of AGA progression necessitates tools that can track microscopic changes over time. İritaş and Özcan [20] highlighted the value of trichoscopy in following treatment response, evidencing significant shifts in hair density and diameter after pharmacological interventions such as minoxidil and finasteride. This enables clinicians to tailor therapies based on real‐time scalp responses rather than relying solely on clinical observation.

An illustrative example is that trichoscopy helps identify early miniaturization of hair follicles before visible thinning appears—a critical window for intervention. Consequently, trichoscopy shifts the management paradigm from reactive to proactive, supporting early diagnosis and timely therapeutic adjustments. This rethinking affirms trichoscopy not merely as a diagnostic tool but as a continuous monitoring system central to personalized AGA care.

### 4.2 Trichoscopy as a Predictive Biomarker Platform in Precision Hair Medicine

As precision medicine gains traction, trichoscopy's role evolves beyond visualization to becoming a predictive biomarker platform. The integration of genomic data and trichoscopic findings allows clinicians to predict disease trajectory and therapeutic outcomes more accurately. Vila‐Vecilla et al. [10] underscored the utility of combining genomic markers with trichoscopic data to stratify patients and personalize treatment strategies, representing a significant leap toward precision hair medicine.

For instance, the 26‐SNP panel described by Gaboardi et al. [14] provides predictive insight into individual responsiveness to anti‐androgen treatments, a vital aspect of tailoring therapy in AGA. When combined with trichoscopy, which quantifies phenotypic hair changes, this genomic‐informed approach enables an integrated assessment of both genetic predisposition and real‐time pathophysiological status.

This dual modality acts as a biomarker platform that not only forecasts treatment response but also monitors biochemical and structural scalp changes. Such synergy exemplifies the potential of trichoscopy as a cornerstone in predictive diagnostics and therapeutic guidance, paving the way for more effective and individualized patient care.

### 4.3 Biological Plausibility of Dermoscopic Predictors

The biological plausibility underlying dermoscopic predictors is well‐established, rooted in recognized pathophysiological changes in AGA. Trichoscopy's ability to detect hair shaft diameter variability and characteristic follicular architecture directly reflects underlying follicular miniaturization and inflammation [24]. Rudnicka et al. [6] were pioneers in correlating these visual markers with histopathological changes, reinforcing the method's biological validity.

Kasumagic‐Halilovic [25] further substantiated that specific trichoscopic features, such as peripilar signs and empty follicular openings, serve as reliable markers of disease severity and activity. Such predictors align biologically with androgen‐mediated follicular miniaturization and perifollicular fibrosis, confirming their diagnostic and prognostic value.

This biological plausibility underpins the clinical confidence in using trichoscopy as a diagnostic criterion. By interpreting dermoscopic patterns, clinicians tap into the molecular and histological dynamics shaping AGA, transforming the scalp examination into a window revealing follicular health in vivo.

### 4.4 Role of Artificial Intelligence and Quantitative Standardization

Artificial intelligence (AI) and quantitative standardization represent the next frontier in enhancing trichoscopy's precision and clinical utility. Zengarini et al. [8] explored computational models that automate image analysis and quantitatively assess scalp and hair parameters, reducing operator bias and improving reproducibility. These advancements allow widespread clinical adoption with consistent and objective trichoscopic evaluations.

For example, AI‐driven algorithms can automatically measure hair density, detect miniaturization patterns, and classify severity stages, enabling faster and more accurate diagnoses. Integration with machine learning also paves the way for predictive analytics, enabling risk stratification and personalized treatment planning based on quantifiable metrics.

The establishment of quantitative standards facilitates cross‐center data comparability, crucial for multicenter clinical trials and longitudinal studies. Thus, AI and standardization do not merely augment the existing method but redefine trichoscopy's role as a robust, scalable biomarker assessment platform in AGA.

### 4.5 Clinical Implications and Suggested Monitoring Strategy

The clinical implications of trichoscopy in AGA extend from diagnosis to monitoring therapeutic efficacy and disease progression. As demonstrated by Kaiser et al. [23], trichoscopy guides clinicians in making informed treatment decisions by visualizing subtle hair and scalp changes that precede gross clinical alterations. For instance, identifying early follicular miniaturization allows adjustment of treatment before significant hair loss occurs.

A practical example is the use of trichoscopy in monitoring minoxidil therapy, where Shukla et al. [26] documented early trichoscopic improvements correlating with clinical hair regrowth, allowing early validation of treatment efficacy. Such monitoring strategies optimize patient compliance and therapeutic outcomes by providing visual evidence of progress or resistance.

Based on current evidence, a suggested monitoring strategy involves baseline trichoscopy at diagnosis, followed by scheduled evaluations every 3–6 months during therapy. This approach facilitates timely detection of treatment response or failure, enabling dynamic modification of hair loss management protocols.

### 4.6 Limitations and Heterogeneity

Despite its numerous advantages, trichoscopy faces limitations, and heterogeneity that must be acknowledged. Feldman et al. [11] pointed out the variability in interpretation and lack of standardized protocols across studies, which confound direct comparisons and meta‐analyses. The heterogeneity in patient populations, disease stages, and treatment modalities further complicates extrapolating results.

Moreover, operator dependency and subjective interpretation can affect trichoscopic findings, emphasizing the need for training and integration of AI for quantitative assessment. Additionally, trichoscopy may not fully capture deeper follicular or molecular changes that require complementary diagnostic modalities.

Recognizing these limitations underscores the importance of standardizing trichoscopic practices and combining them with molecular diagnostics. Such integrative approaches hold promise to overcome current barriers and enhance the method's reliability and clinical applicability.

## 5 Conclusion

In summary, trichoscopy has transformed the landscape of androgenetic alopecia management, evolving from a diagnostic adjunct to a pivotal tool in personalized hair medicine. Its ability to non‐invasively visualize microscopic hair and scalp changes provides valuable biological and clinical insights, underpinning early diagnosis and treatment monitoring.

The integration of genomic biomarkers and AI‐driven quantitative analysis holds great promise in refining precision medicine approaches, enabling tailored therapies based on predictive and dynamic assessments. Nonetheless, addressing current limitations through standardization and complementary diagnostics remains imperative.

Future research should focus on validating trichoscopy‐driven algorithms and expanding biomarker platforms to fully harness its potential in AGA management. This will ensure that trichoscopy maintains its essential role in delivering precise, evidence‐based care for patients with androgenetic alopecia.

## Author Contributions

M.M. was responsible for data curation, conceptualization, and original draft preparation; N.K.J. contributed to conceptualization and methodology; D.H. contributed to conceptualization and supervision; I.B.P. contributed to conceptualization and supervision. All the authors read and approved the manuscript.

## Funding

The authors have nothing to report.

## Ethics Statement

The authors have nothing to report.

## Conflicts of Interest

The authors declare no conflicts of interest.

## Data Availability

Data sharing is not applicable to this article as no new data were created in this study.

## References

[1] A. K. Gupta , T. Wang , V. Economopoulos , et al., “Epidemiological Landscape of Androgenetic Alopecia in the US: An All of US Cross‐Sectional Study,” PLoS One 20, no. 2 (2025): e0319040, 10.1371/journal.pone.0319040. 40014580 PMC11867384

[2] K. D. Kaufman , E. A. Olsen , D. Whiting , et al., “Finasteride in the Treatment of Men With Androgenetic Alopecia. Finasteride Male Pattern Hair Loss Study Group,” Journal of the American Academy of Dermatology 39, no. 4 (1998): 578–589, 10.1016/s0190-9622(98)70007-6. 9777765

[3] E. A. Olsen , F. E. Dunlap , T. Funicella , et al., “A Randomized Clinical Trial of 5% Topical Minoxidil Versus 2% Topical Minoxidil and Placebo in the Treatment of Androgenetic Alopecia in Men,” Journal of the American Academy of Dermatology 47, no. 3 (2002): 377–385, 10.1067/mjd.2002.124088. 12196747

[4] A. Kuczara , A. Waśkiel‐Burnat , A. Rakowska , et al., “Trichoscopy of Androgenetic Alopecia: A Systematic Review,” Journal of Clinical Medicine 13, no. 7 (2024): 1962, 10.3390/jcm13071962. 38610726 PMC11012765

[5] A. K. Gupta , M. Talukder , and G. Williams , “Comparison of Oral Minoxidil, Finasteride, and Dutasteride for Treating Androgenetic Alopecia,” Journal of Dermatological Treatment 33, no. 7 (2022): 2946–2962, 10.1080/09546634.2022.2109567. 35920739

[6] L. Rudnicka , M. Olszewska , A. Rakowska , E. Kowalska‐Oledzka , and M. Slowinska , “Trichoscopy: A New Method for Diagnosing Hair Loss,” Journal of Drugs in Dermatology: JDD 7, no. 7 (2008): 651–654. 18664157

[7] N. U. Saqib , Y. J. Bhat , I. H. Shah , et al., “Assessment, Reliability, and Validity of Trichoscopy in the Evaluation of Alopecia in Women,” International Journal of Women's Dermatology 7, no. 4 (2021): 458–465, 10.1016/j.ijwd.2021.02.002. PMC8484948 34621959

[8] C. Zengarini , N. Curti , S. Cedirian , et al., “Trichoscopy and Computational Models for Hair and Scalp Disorders: Image Analysis, Quantification, and Clinical Integration,” Applied Sciences 16, no. 7 (2026): 3199, 10.3390/app16073199.

[9] D. Van Neste , “Exhaustive Analysis of Scalp Hair Regression: Subjective and Objective Perception From Initial Hair Loss to Severe Miniaturisation and Drug‐Induced Regrowth,” Plastic and Aesthetic Research 8 (2021): 16, 10.20517/2347-9264.2020.220.

[10] L. Vila‐Vecilla , V. Russo , and G. T. de Souza , “Genomic Markers and Personalized Medicine in Androgenetic Alopecia: A Comprehensive Review,” Cosmetics 11, no. 5 (2024): 148, 10.3390/cosmetics11050148.

[11] P. R. Feldman , P. Gentile , C. Piwko , et al., “Hair Regrowth Treatment Efficacy and Resistance in Androgenetic Alopecia: A Systematic Review and Continuous Bayesian Network Meta‐Analysis,” Frontiers in Medicine 9 (2023): 998623, 10.3389/fmed.2022.998623. 36755885 PMC9900126

[12] R. M. Trüeb , V. M. L. Jolliffe , A. F. Régnier , et al., “Precision Medicine and the Practice of Trichiatry: Adapting the Concept,” Skin Appendage Disorders 5, no. 6 (2019): 338–343, 10.1159/000500364. 31799259 PMC6883441

[13] H. Johnson , D. Huang , A. K. Clift , et al., “Effectiveness of Combined Oral Minoxidil and Finasteride in Male Androgenetic Alopecia: A Retrospective Service Evaluation,” Cureus 17, no. 1 (2025): e77549, 10.7759/cureus.77549. 39958004 PMC11829753

[14] H. Gaboardi , V. Russo , L. Vila‐Vecilla , V. Patel , and G. T. De Souza , “26‐SNP Panel Aids Guiding Androgenetic Alopecia Therapy and Provides Insight Into Mechanisms of Action,” Cosmetics 12, no. 5 (2025): 190, 10.3390/cosmetics12050190.

[15] A. Rossi and G. Caro , “Efficacy of the Association of Topical Minoxidil and Topical Finasteride Compared to Their Use in Monotherapy in Men With Androgenetic Alopecia: A Prospective, Randomized, Controlled, Assessor Blinded, 3‐Arm, Pilot Trial,” Journal of Cosmetic Dermatology 23 (2024): 502–509, 10.1111/jocd.15953. 37798906

[16] M. N. Patel , N. Patel , and A. Merja , “Advancing Hair Regrowth Assessment: Development and Standardization of Methods for Evaluating New Hair Growth, Hair Keratin Levels, and Scalp Health in Androgenetic Alopecia Patients,” Cureus 17, no. 2 (2025): e79859, 10.7759/cureus.79859. 40170743 PMC11958839

[17] R. Sinclair , J. Sicinska , L. Bokhari , and M. Kasprzak , “Sublingual Minoxidil Increases Fibre Diameter in Male Androgenetic Alopecia: A Proxy for Reversal of Hair Follicle Miniaturization,” Clinical and Experimental Dermatology 50, no. 7 (2025): 1362–1365, 10.1093/ced/llaf012. 39774750

[18] A. Waśkiel‐Burnat , A. Rakowska , M. Sikora , M. Olszewska , and L. Rudnicka , “Alopecia Areata Predictive Score: A New Trichoscopy‐Based Tool to Predict Treatment Outcome in Patients With Patchy Alopecia Areata,” Journal of Cosmetic Dermatology 19 (2020): 746–751, 10.1111/jocd.13064. 31301100

[19] F. R. Dias , S. S. Yong , H. FitzGerald , R. D. Sinclair , and B. Bhoyrul , “Expanding the Therapeutic Landscape of Minoxidil for Androgenetic Alopecia: Topical, Oral and Sublingual Formulations,” Frontiers in Pharmacology 16 (2026): 1718208, 10.3389/fphar.2025.1718208. 41695992 PMC12898826

[20] S. Y. İritaş and D. Özcan , “The Value of Trichoscopy in the Follow‐Up of Treatment Response in Patients With Androgenetic Alopecia,” Dermatology Practical and Conceptual 14, no. 1 (2024): e2024001, 10.5826/dpc.1401a1. 38048259 PMC10868879

[21] Y. Ovcharenko , K. Khobzei , and N. Lortkipanidze , “Androgenetic Alopecia,” in Psychotrichology, ed. Y. Ovcharenko , A. Lyakhovitsky , and M. Jafferany (Springer, 2025), 10.1007/978-3-031-64083-4_9.

[22] S. Umar , B. H. Tan , and P. K. Shitabata , “Perifollicular Inflammation and Fibrosis in Androgenetic Alopecia: Implications for Diagnosis and Treatment ‐ A Comparative Histopathologic and Clinical Study With Normal‐Appearing Scalp,” Clinical, Cosmetic and Investigational Dermatology 19 (2026): 548520, 10.2147/CCID.S548520. 41858594 PMC12997279

[23] M. Kaiser , R. Abdin , S. I. Gaumond , N. T. Issa , and J. J. Jimenez , “Treatment of Androgenetic Alopecia: Current Guidance and Unmet Needs,” Clinical, Cosmetic and Investigational Dermatology 16 (2023): 1387–1406, 10.2147/CCID.S385861. 37284568 PMC10239632

[24] B. Olędzka , M. Piątkiewicz , and A. Rakowska , “The Usefulness of Trichoscopy in the Diagnosing of Fibrosing Alopecia in Pattern Distribution,” Forum Dermatologicum 11, no. 1 (2025): 34–36, 10.5603/fd.104788.

[25] E. Kasumagic‐Halilovic , “Trichoscopic Findings in Androgenetic Alopecia,” Medical Archives 75, no. 2 (2021): 109–111, 10.5455/medarh.2021.75.109-111. 34219869 PMC8228579

[26] N. Shukla , C. S. Jaiswal , J. Nagwanshi , D. Dandotiya , and R. Sharma , “Early Trichoscopic Changes of Minoxidil Therapy in Male Pattern Hair Loss: A Prospective Cohort Study,” Journal of Pharmacy and Bioallied Sciences 18, no. 2 (2026): 108–111, 10.4103/jpbs.jpbs_1711_24. 42005482 PMC13086379

